# The COMiT’ID Study: Developing Core Outcome Domains Sets for Clinical Trials of Sound-, Psychology-, and Pharmacology-Based Interventions for Chronic Subjective Tinnitus in Adults

**DOI:** 10.1177/2331216518814384

**Published:** 2018-11-29

**Authors:** Deborah A. Hall, Harriet Smith, Alice Hibbert, Veronica Colley, Haúla F. Haider, Adele Horobin, Alain Londero, Birgit Mazurek, Brian Thacker, Kathryn Fackrell, for the Core Outcome Measures in Tinnitus (COMiT) initiative

**Affiliations:** 1 National Institute for Health Research Nottingham Biomedical Research Centre, Ropewalk House, UK; 2 Hearing Sciences, Division of Clinical Neuroscience, School of Medicine, University of Nottingham, UK; 3 Nottingham University Hospitals National Health Service Trust, Queens Medical Centre, UK; 4 University of Nottingham Malaysia, Semeniyh, Malaysia; 5 ENT Department, Hospital Cuf Infante Santo—Nova Medical School, Lisbon, Portugal; 6 Service ORL et CCF, Consultation Acouphène et Hyperacousie, Hôpital Européen G. Pompidou, Paris, France; 7 Tinnitus Center, Charite University Hospital, Berlin, Germany

**Keywords:** assessment, patient-reported outcome measures, treatment effectiveness, stakeholder agreement

## Abstract

Subjective tinnitus is a chronic heterogeneous condition that is typically managed using intervention approaches based on sound devices, psychologically informed therapies, or pharmaceutical products. For clinical trials, there are currently no common standards for assessing or reporting intervention efficacy. This article reports on the first of two steps to establish a common standard, which identifies what specific tinnitus-related complaints (“outcome domains”) are critical and important to assess in all clinical trials to determine whether an intervention has worked. Using purposive sampling, 719 international health-care users with tinnitus, health-care professionals, clinical researchers, commercial representatives, and funders were recruited. Eligibility was primarily determined by experience of one of the three interventions of interest. Following recommended procedures for gaining consensus, three intervention-specific, three-round, Delphi surveys were delivered online. Each Delphi survey was followed by an in-person consensus meeting. Viewpoints and votes involved all stakeholder groups, with approximately a 1:1 ratio of health-care users to professionals. “Tinnitus intrusiveness” was voted in for all three interventions. For sound-based interventions, the minimum set included “ability to ignore,” “concentration,” “quality of sleep,” and “sense of control.” For psychology-based interventions, the minimum set included “acceptance of tinnitus,” “mood,” “negative thoughts and beliefs,” and “sense of control.” For pharmacology-based interventions, “tinnitus loudness” was the only additional core outcome domain. The second step will next identify how those outcome domains should best be measured. The uptake of these intervention-specific standards in clinical trials will improve research quality, enhance clinical decision-making, and facilitate meta-analysis in systematic reviews.

## Introduction

Evidence-based clinical practice relies on findings from high-quality clinical trials to test whether any individual intervention is beneficial and safe for patients. The selection and reporting of outcomes in a clinical trial is one of the most critical factors in any assessment of the effectiveness of an intervention (Noble, 2001). Yet, a recent systematic review (Hall et al., 2016) found wide diversity in the outcomes assessed and reported in clinical trials of tinnitus interventions, with no single outcome being selected across all studies. Most studies failed to clearly define the tinnitus-related concepts of interest (Hall et al., 2016).

In a series of recent articles, arguments have built on earlier calls (such as Landgrebe et al., 2012) for engaging the international tinnitus community in addressing these important methodological issues (Hall, 2017; Hall et al., 2015, 2016; Londero & Hall, 2017). Outcome diversity could be reduced through recommendations for a minimum reporting standard for outcomes to be assessed and reported in all clinical trials of tinnitus interventions. International initiatives are now actively promoting minimum reporting standards across clinical trials in all medical specialties. The Core Outcome Measures in Effectiveness Trials (COMET) initiative and COnsensus-based Standards for the selection of health Measurement Instruments initiative are two prominent examples that promote robust methodologies for developing minimum standards for clinical trial outcomes (see Prinsen et al., 2016; Williamson et al., 2017). The overall program of work advocated by these initiatives clearly separates the process of specifying *what* to measure (outcome domains) from that of *how* to measure it (outcome measurement instruments) into two discrete and sequential steps. This ordering is intentional so as to encourage open-minded choices about which therapeutic targets are the most important to the greatest number of stakeholders and to reduce potential bias toward selecting measurement instruments simply on the basis of their popularity or accessibility.

This article reports the methods and findings from the first step in this process which considers only *what* tinnitus-related outcome domains should form the common standard, not yet *how* they should be measured. Specifically, an outcome domain is any distinct element of tinnitus (i.e., a patient complaint) which could be assessed to determine whether an intervention has worked. A minimum reporting standard for tinnitus clinical trials that recommends what the core outcome domains should be and how they should be measured would bring distinct advantages. First, it would enable robust conclusions to be made about the effectiveness of tinnitus interventions, by enabling meta-analysis (i.e., combining the findings from different studies reporting the effectiveness of the same tinnitus intervention) and by supporting direct comparisons of findings across different therapeutic approaches (Clarke & Williamson, 2016). Second, it would reduce the risk of outcome reporting bias. This refers to when researchers select a subset of the original measured outcomes for publication based on the findings obtained after study completion (Dwan, Gamble, Williamson, & Kirkham, 2013) or when researchers simply fail to report prespecified outcomes (Smith, Clarke, Williamson, & Gargon, 2015). Finally, if people with chronic subjective tinnitus were actively involved in making the decisions about the common set of outcomes, then a minimum reporting standard would also bring face validity to outcome selection. This would ensure that outcomes and subsequent conclusions are relevant to the end users. Overall, a minimum reporting standard would reduce wasteful research (Chalmers & Glaziou, 2009), promoting international collaboration and knowledge gain. A minimum standard, or “core set,” is not restrictive; it does not necessarily exclude other outcomes from being assessed in individual trials; it is simply a way to reduce diversity and enable a basis of comparison from one study to another.

For chronic subjective tinnitus, the process of identifying a core set of outcome domains, to be commonly assessed when evaluating an intervention, is somewhat complicated by the fact that there are different intervention approaches (Baguley, McFerran, & Hall, 2013). Chronic subjective tinnitus is characterized by not having a readily identifiable single cause, physiological or otherwise, and no permanent cure through, for example, surgery. Therefore, at this point in time, interventions rely on managing the symptoms of tinnitus, either by reducing the perception of the tinnitus sounds or minimizing their impact on the individual’s life. Most existing management options can be split broadly into two families: sound- and psychology-based approaches (e.g., Fuller et al., 2017; Tunkel et al., 2014). Sound-based approaches include electronic devices that increase audibility associated with any comorbid hearing loss that exacerbates the tinnitus (e.g., hearing aids, cochlear implants) or produce therapeutic sounds to mask or distract from tinnitus (e.g., wearable sound generators, mobile phone applications, or the radio; Hoare, Searchfield, El Refaie, & Henry, 2014; Ramakers, van Zon, Stegeman, & Grolman, 2015). Psychology-based approaches can include talking-based methods to help people deal with how tinnitus makes them feel and behavior change methods to empower them with ways of managing it (e.g., cognitive behavioral therapy and mindfulness; Thompson, Hall, Walker, & Hoare, 2017). In addition, although there are currently no medications approved specifically for tinnitus, a variety of licensed drugs have been used off-label to treat the condition (Elgoyhen & Langguth, 2010). Licensed medication is also prescribed to alleviate common complaints associated with tinnitus such as depressive symptoms, anxiety, and sleep difficulties. Therefore, pharmacology-based approaches for tinnitus are an established medical practice. This informed our decision to consider pharmacology-based interventions as a third family of tinnitus management options for the purpose of this project. Other therapeutic approaches have been evaluated in research settings such as neuromodulation therapies (e.g., repetitive transcranial magnetic stimulation, epidural stimulation) and nonprescription food supplements (e.g., Gingko biloba, zinc supplements), but national standard intervention options offered to those people with tinnitus seeking professional advice essentially rely upon sound devices, talking therapies, and drug products at this time. Each of the family of intervention approaches introduced—sound, psychology, and pharmacology—has a different therapeutic rationale, and so it should not be assumed that intervention success should be assessed using the same set of outcome domains in all cases.

The project objective was therefore to select the outcome domains for all future early-phase clinical trials of sound-, psychology-, and pharmacology-based interventions that target the intervention or management of chronic subjective tinnitus in adults, regardless of the specific nature of the intervention or mode of administration. To achieve this, three separate development studies were conducted in parallel, one for each family of interventions. A wide range of stakeholders with expert knowledge of tinnitus were engaged in this development process, which was intended to ensure that each minimum reporting standard would represent the views and interests of all end users, to increase the likelihood that the wider community would agree with the outcomes selected, and to enhance the prospect of endorsement and implementation of the recommendations. While the primary objective was to develop a Core Outcome Domain Set for the design of clinical trials in chronic subjective tinnitus, these standards should be considered for use in other research designs, and notably in systematic reviews.

## Methods

**Figure 1.**
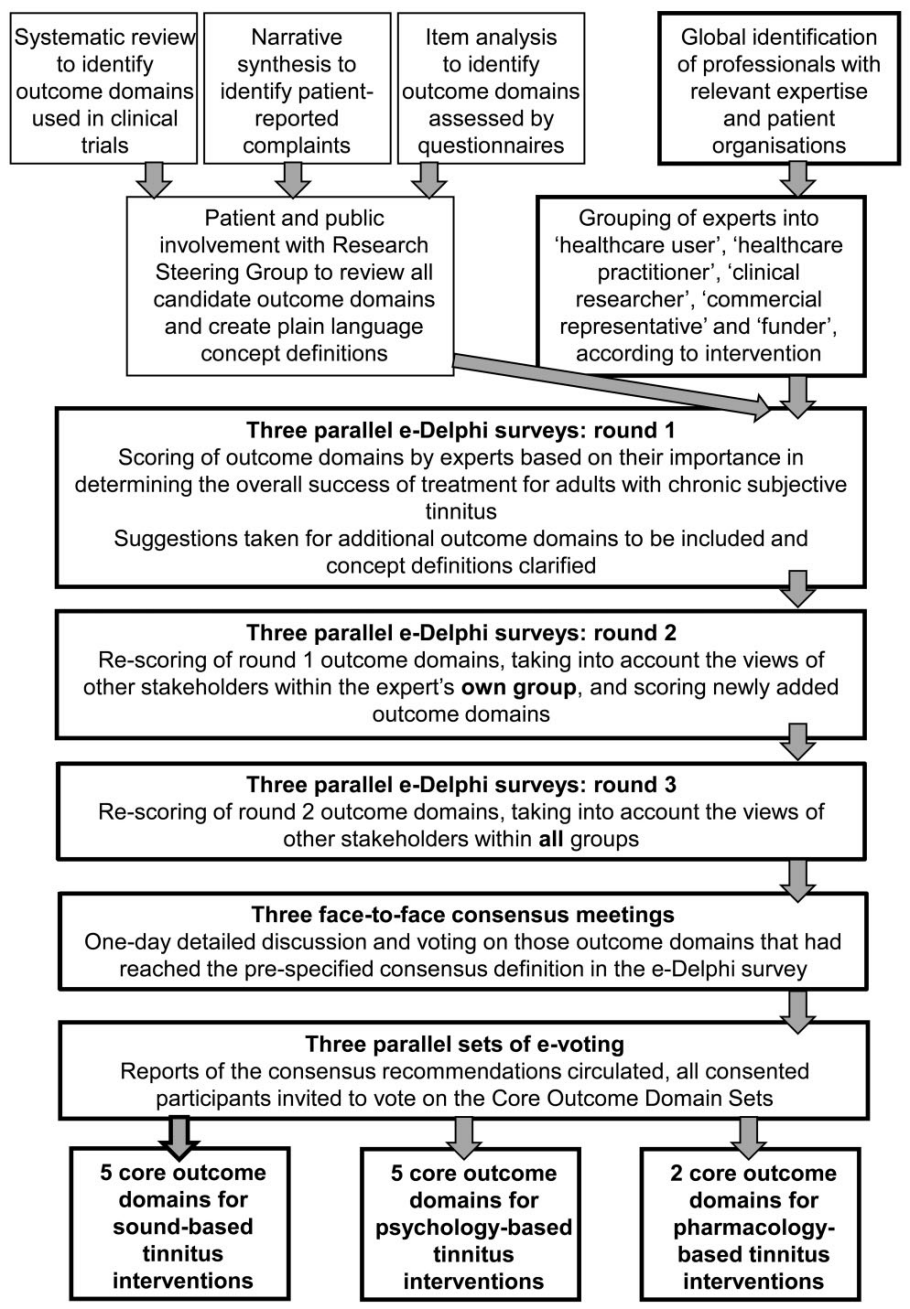
Schematic overview for the overall project including three parallel e-Delphi surveys, the corresponding face-to-face meetings to establish the Core Outcome Domain Set recommendations, and stakeholder voting for each intervention-specific strand. The phases reported in this article are outlined in bold.

An overview of the development process is provided in Figure 1, and the phases reported in this article are outlined in bold. This article reports the three phases of the project to establish the Core Outcome Domain Set for sound-, psychology-, and pharmacology-based interventions, respectively. These three phases comprised the Core Outcome Measures in Tinnitus International Delphi (COMiT’ID) study. The first phase comprised three Delphi surveys to prioritize outcome domains for each family of interventions. These used three rounds of questions and were delivered online (e-Delphi) to engage a large number of international stakeholders. The second phase involved structured face-to-face meetings with a smaller subset of stakeholders, allowing for in depth conversation to reduce the priority list to a core set. The third and final phase widened input to all consented stakeholders inviting them to vote on the intervention-specific recommendations.

This article adheres to the guidelines for the methodology (Williamson et al., 2017) and reporting (Kirkham et al., 2016) of Core Outcome Domain Set development studies. The study design was approved by the West Midlands—Solihull Research Ethics Committee and Health Research Authority (ref: 17/WM/0095, March 2017), and the protocol is published (Fackrell et al., 2017). Informed consent was given through the online e-Delphi process, and written consent was given for participation at the consensus meeting.

### Participants

Five types of stakeholders were targeted to participate in the prioritization process; health-care users, health-care practitioners, clinical researchers, commercial representatives, and funders. Individuals were invited to participate using a purposive sampling approach, with recruitment methods and eligibility criteria detailed in the protocol (Fackrell et al., 2017). All participants were required to be at least 18 years old and able to read, understand, and complete surveys in English.

### e-Delphi Surveys

The overall e-Delphi project had a minimum target sample size of 260 participants across the three e-Delphi surveys, aiming for a 50:50 balance of health-care users and professionals and aiming for the minimum targets to be maintained at Round 3. Commercial representatives and funders were pooled in the same group, as per protocol (Fackrell et al., 2017). Participants could complete one, two, or three e-Delphi surveys, depending on his or her level of experience and specific knowledge. To promote retention of participants, each round was open for a short time, and the time between rounds was kept to a minimum. Response rates were regularly monitored, automated reminders were issued from the survey software, and personalized e-mail reminders were sent to target individuals who were yet to complete the round.

A modified e-Delphi technique was used, presenting participants with a “long list” of candidate outcome domains each with a plain language concept definition. As per protocol (Fackrell et al., 2017), three information sources were used to create the long list: a systematic review of outcome domains used in clinical trials of tinnitus interventions (Hall et al., 2016), a narrative synthesis of research evidence for patient-reported complaints of tinnitus (Hall et al., 2018a), and a thematic analysis of all items in 23 of the most common tinnitus questionnaires to identify outcome domains assessed (unpublished). The long list initially comprised 124 outcome domains that were reduced to 66 through a series of health-care user-led decisions, removing or combining domains with overlapping concepts, not specific to tinnitus, or associated with measurement of the construct (Fackrell et al., 2017; Smith et al., 2018).

All three e-Delphi surveys presented the same list of 66 candidate outcome domains over three rounds, but outcome domains were scored from the perspective of the intervention in question. All outcome domains were retained from one round to the next so that participants were free to change their scores across rounds. In Round 1, participants were invited to propose additional outcome domains. Two members of the Study Management Team reviewed these proposals, and new items sufficiently distinct from existing candidate outcomes were added to the list within the most relevant category, each with a corresponding plain language concept definition, to be scored by all participants in Rounds 2 and 3.

To engage with international stakeholders, the scoring process for the three e-Delphi surveys was managed online using DelphiManager software maintained by the COMET initiative at the University of Liverpool (see Williamson et al., 2017). At each round, participants were asked to think about the importance of each of the 66 tinnitus outcome domains and indicate how important it is to measure when deciding if a sound-, psychology-, or pharmacology-based tinnitus intervention is working, respectively. Pharmacology-based interventions specifically excluded herbal remedies and dietary supplements. Participants scored each outcome domain using the GRADE scale of 1 to 9 (Guyatt et al., 2011). Scoring used a Likert scale with additional interpretation categories; 1 to 3 indicated that the domain was *not important*, 4 to 6 indicated it was *important but not critical*, and 7 to 9 indicated that it was *critically important* in deciding whether a tinnitus intervention is effective. “Unable to score” was always an option, and there were open-text boxes for adding comments. In the subsequent rounds, participants were presented their previous score and numerical and graphical feedback on the distribution of scores for each outcome domain. The purpose of Round 2 was to enable participants to reflect on their scores in light of the distribution of scores from their own stakeholder group and to score the outcomes again. The purpose of Round 3 was to enable participants to reflect on their scores in light of the distribution of scores from all stakeholder groups and to score the outcomes again. Participant data in which less than 40% of outcome domains had been scored were removed from the results presented at subsequent rounds as per protocol (Fackrell et al., 2017).

During the e-Delphi surveys, the scoring from each stakeholder group was kept separate to ensure the interests of all relevant parties were reflected. From Round 3, a recommendation for inclusion was defined as at least 70% of the participants in all stakeholder groups scoring 7 to 9 and fewer than 15% in any stakeholder group scoring 1 to 3.

### Face-to-Face Meetings

Participants who responded to ≥ 90% of the outcome domains in Round 3 of the e-Delphi survey were invited to participate in a meeting. Participants could only attend one of the three meetings, irrespective of how many e-Delphi surveys he or she had completed. There was an expected 50:50 balance of health-care users and professionals, including non-U.K. participants.

Each meeting lasted for 1 day, and the discussion was semistructured according to the nominal group technique (Harvey & Holmes, 2012) using a blend of whole-group and subgroup discussions, sharing of ideas and voting techniques. Participants were encouraged to voice their opinions, with a prerequisite that all were equal and that every contribution was valid. The meeting was led by an impartial facilitator, as well as two “table hosts” whose role it was to keep a focus and to encourage all participants to contribute to the subgroup discussions and two “patient buddies” to support the health-care users. The meetings aimed to reduce the list of candidate domains identified as important during the e-Delphi to just those to be included in the Core Outcome Domain Sets. Initially, the meeting was focused on identifying and voting on outcomes to be set aside, which should *not* be included in the final set. Following this, the discussions were focused on whether each of the remaining outcomes should be included in the Core Outcome Domain Set. All decisions about the final selections were voted in a first round with “agree”, “disagree”, or “unsure” as response options. Scores were expressed as percentages using a real-time, anonymized voting system (CLiKAPAD Ltd, East Sussex, UK).

For the face-to-face meetings, the criterion for consensus was at least 70% agreement on each vote, but here, all participants were treated equally, and so votes were not separated by stakeholder group. Votes that did not reach consensus for either “agree” or “disagree” were reopened for discussion by the facilitator. In the final votes, “unsure” responses were discouraged, and only those outcome domains that reached 70% agree were included in the Core Outcome Domain Set, with failure to reach a consensus resulting in the outcome domain being set aside.

### Final Voting

All e-Delphi survey study participants were given an opportunity to submit an e-mail vote on the final Core Outcome Domain Set for each intervention category, with the response options “agree”, “disagree”, or “have no strong opinion and will go along with the majority.”

### Changes From Protocol

Two changes to the protocol (Fackrell et al., 2017) were made by the Study Management Team after data analysis of the e-Delphi Round 3. It was originally planned to discuss all 66 + outcome domains at the face-to-face meetings. But instead, only those outcome domains reaching consensus (70% scoring 7–9 in all stakeholder groups) were discussed at the face-to-face meeting to allow participants to focus on discriminating between those outcome domains most likely to form the minimum reporting standard. Second, the protocol had placed no upper limit on the number of outcome domains within any Core Outcome Domain Set. However, it was decided to restrict the maximum number to six to ensure practicality of use in a clinical trial. These two procedural changes were proposed to the participants in each face-to-face meeting and received majority approval. For the sound-, psychology-, and pharmacology-based meetings, respectively, votes were as follows: “agree” = 89%, 79%, 94%; “disagree” = 5%, 11%, 6%; and “unsure” = 5%, 11%, 0%.

## Results

### Participants

Overall, there were 719 participants across the three development studies. Round 1 was completed by 670 participants, Round 2 by 586 (87.5%) and Round 3 by 533 (79.6%) giving an acceptable retention rate (Table 1). The e-Delphi surveys were successful in maintaining their minimum targets at Round 3.

**Table 1.** Flow of Participants Through the Studies Reporting the Recruitment Target That Was Prespecified in the Protocol (Fackrell et al., 2017), the Number of Participants Who Consented and Subsequently Completed Each Round of the e-Delphi Survey, and the Number Attending the Face-to-Face Consensus Meetings.

Stakeholder group	Recruitment target (min–max)	Consented	e-Delphi Round 1	e-Delphi Round 2	e-Delphi Round 3	Retention rate (%)	Meeting
**Sound-based interventions**							
Health-care user	60–90	199	182	160	142	78.0	10
Health-care practitioner	20–30	79	70	60	57	81.4	5
Clinical researchers	20–30	36	35	35	34	97.1	3
Commercial reps and funders	20–30	24	21	19	19	90.5	1
**Psychology-based interventions**							
Health-care user	40–60	118	114	97	89	78.1	10
Health-care practitioner	20–30	63	61	57	50	82.0	4
Clinical researchers	20–30	39	39	37	36	92.3	5
Commercial reps and funders^a^	N/A	4	4	4	3	N/A	N/A
**Pharmacology-based interventions**							
Health-care user	30–60	67	62	48	41	66.1	6
Health-care practitioner	10–20	51	47	40	37	78.7	5
Clinical researchers	10–20	20	17	14	13	76.5	2
Commercial reps and funders	10–20	19	18	15	12	66.7	3
Total		719^b^	670	586	533^c^	79.6	54

*Note*. The minimum value in the recruitment target range was expected to be maintained through to Round 3. Retention rate was calculated from Round 1 to Round 3.

a Note that this stakeholder group was not purposively sampled for the psychology-based intervention survey, and so 70% agreement among those participants was not required for consensus decision-making.

b Note some individuals consented to participate in more than one study, and so when those duplicates have been accounted for, the 719 comprises 641 unique individuals.

c For those participating in more than one study, when duplicates have been accounted for, the 533 comprises 472 unique individuals.

**Figure 2.**
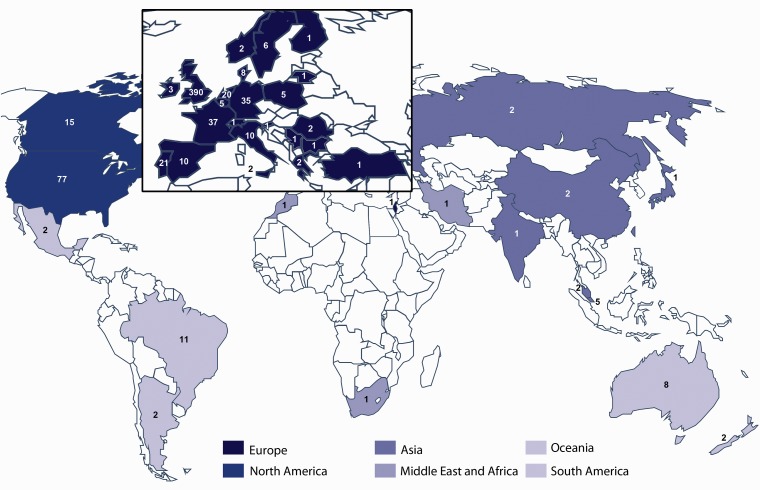
World map illustrating the geographical dispersal of all consenting participants across studies. Regional groupings are inspired by the World Health Organization (WHO) regional classification. To reflect English language-speaking countries, the WHO region of the Americas was separated into North and South America. Similarly, Australia and New Zealand were considered separately from the Western Pacific region, as Oceania. Country-specific data indicate only two participants in Africa, and so this was combined with countries in the WHO Eastern Mediterranean region to create the Middle East and Africa region.

Figure 2 illustrates the geographical distribution, and Table 2 shows the age distribution of consented participants in the three e-Delphi surveys. All continents were represented, although there was an expected bias toward English-speaking countries and Europe. The tinnitus population was well represented, with about two thirds of all health-care users aged older than 50 years. There were 31 journal editors. Health-care practitioners predominantly identified themselves as audiologists, hearing therapists, otolaryngologists, and psychologists, with a small number of neurologists, psychiatrists, general practitioners, and physical therapists.

**Table 2.** Age of All Consented e-Delphi Participants Split by Stakeholder Group.

Stakeholder group	18–29 years	30–49 years	50–69 years	70–89 years	Total
Health-care users	22 (6)	98 (26)	218 (57)	46 (12)	384
Health-care practitioners	7 (3)	87 (42)	108 (52)	4 (2)	206
Clinical researchers	7 (9)	43 (52)	31 (38)	1 (1)	82
Commercial reps and funders	1 (2)	24 (51)	21 (45)	1 (2)	47
All stakeholder groups	37 (5)	252 (35)	378 (53)	52 (7)	719

*Note*. Percentages within each stakeholder group are reported in parentheses.

For each face-to-face meeting, there was a maximum of 20 places, but not all places were filled despite sending invitation reminders. Nineteen participants met to discuss the sound-based Core Outcome Domain Set, 19 met for the psychology-based Core Outcome Domain Set, and 16 met for the pharmacology-based Core Outcome Domain Set. The distribution of participants across stakeholder groups is shown in Table 1. Each meeting comprised relevant health-care practitioners from each major clinical specialty. Furthermore, all meetings had representation from outside the United Kingdom (sound-based meeting: France, Germany, Netherlands; psychology-based meeting: Belgium, France; pharmacology-based meeting: Belgium, Brazil, France, Germany, Portugal, Spain, Switzerland).

### e-Delphi Surveys

Sixty-six outcome domains were presented in Round 1 of the e-Delphi survey (Supplementary File 1). Overall, 213 feedback comments about potential additional outcome domains were made during Round 1. From this feedback, the Study Management Team added seven new outcome domains to all three e-Delphi surveys (Supplementary File 1). From the sound-based survey feedback, additional outcome domains were “device usage” and “frequency of occurrence of tinnitus episodes.” From the psychology-based feedback, they were “guilt,” “monitoring,” “self-harm,” and “teeth clenching”; and for the pharmacology-based feedback, they were “frequency of occurrence of tinnitus episodes” and “pharmacodynamics.” Comments also led to revised wording of the original plain language concept definitions for seven outcome domains, which were changed for the subsequent rounds of all three e-Delphi surveys (Supplementary File 1). Of the remaining feedback comments, more than one third of these suggestions duplicated concepts in the original list (38%), while others considered items that had been excluded at the stage of preparing the long list because they were multidomain concepts (12%), described a comorbidity (12%), or were more associated with how to measure (12%).

Following completion of Round 3, 47 of the 68 candidate outcome domains for the sound-based Core Outcome Domain Set were ruled out because they did not meet the prespecified consensus definition. For the psychology-based Core Outcome Domain Set, 46 of the 70 candidate outcome domains were ruled out, and for the pharmacology-based Core Outcome Domain Set, 51 of the 68 candidate outcome domains were ruled out (Supplementary File 2). The remaining outcome domains are reported in Table 3. These were all considered important and critical according to the consensus definition for the e-Delphi survey.

**Table 3.** All Outcome Domains That Reached the Prespecified Consensus Definition Based on the e-Delphi Round 3 Voting.

Sound-based interventions	Psychology-based interventions	Pharmacology-based interventions
Ability to ignore	Ability to ignore	Ability to ignore
Ability to relax	**Acceptance of tinnitus**	Adverse reaction
Acceptance of tinnitus	Annoyance	Annoyance
Annoyance	Anxiety	Anxiety
Anxiety	Catastrophizing	Concentration
Concentration	Concentration	Confusion
Conversations	Coping	Coping
Coping	Depressive symptoms	Depressive symptoms
Depressive symptoms	Difficulties getting to sleep	Difficulties getting to sleep
Difficulties getting to sleep	Fear	Impact on individual activities
Frequency of occurrence of tinnitus episodes	Helplessness (lack of control)	Impact on social life
Helplessness (lack of control)^a^	Impact on individual activities	Impact on work
Impact on individual activities	Impact on relationships	Quality of sleep
Impact on social life	Impact on social life	**Tinnitus intrusiveness**
Impact on work	Impact on work	**Tinnitus loudness**
Listening	Irritable	Tinnitus unpleasantness
Quality of sleep	**Mood**	Treatment satisfaction
Tinnitus awareness	**Negative thoughts/beliefs**	
Tinnitus intrusiveness	Quality of sleep	
Tinnitus unpleasantness	**Sense of control**	
Treatment satisfaction	Suicidal thoughts	
	**Tinnitus intrusiveness**	
	Tinnitus-related thoughts	
	Worries/concerns	

*Note*. These outcomes were agreed to be important and critical for deciding whether or not an intervention for tinnitus is working. Outcome domains highlighted in bold font are those that were recommended in the final Core Outcome Domain Sets.

a Note that the concept “helplessness” was replaced by “sense of control” during the face-to-face meeting discussion on sound-based interventions, and so the recommendation is for “sense of control.”

### Face-to-Face Meetings

#### Sound-based outcome domains

Twenty-one outcome domains were taken to the face-to-face meeting with the goal to restrict the maximum number to six (Table 3). During the discussion of the outcome domain “helplessness (lack of control),” participants asked the facilitator to share the Round 3 scores for the discarded domain “sense of control” as they felt the two concepts were somewhat similar but that “sense of control” would be preferable due to its more positive phrasing and thus fewer negative connotations. From the e-Delphi sound-based survey, the Round 3 scores (i.e., score 7–9) for “sense of control” had just missed the consensus definition (health-care users = 84.5%, health-care practitioners = 87.7%, clinical researchers = 64.7%, and commercial representatives and funders = 94.7%). The proposal for “sense of control” to replace “helplessness” in the further discussion was put to vote and agreed by 95% of participants (see Table 4 and Supplementary File 3). After voting, five outcome domains met the consensus definition for inclusion in the Core Outcome Domain Set for early-phase clinical trials of sound-based interventions: “ability to ignore,” “concentration,” “sense of control,” “quality of sleep,” and “tinnitus intrusiveness.” Meeting votes and reasons supporting their inclusion are described in Table 4. Supplementary File 3 gives reasons for setting aside the remaining 17 outcome domains.

**Table 4.** Meeting Votes and Comments in Favor and Against Explaining the Reasons for Recommending the Five Outcome Domains for Evaluating Sound-Based Interventions.

Outcome domains	Vote	Comments in favor	Comments against
Ability to ignore	89	• Linked to, but more relevant than, “tinnitus annoyance” • Considered one of the primary objectives for using a sound therapy	*No strong views expressed*
Concentration	74	• Relevant to many different aspects of life • The group recommended that the “measure of concentration” should include a question about conversations.	• Narrowly focussed • Already encompassed by “ability to ignore” • Some participants stated this did not affect them personally.
Sense of control (replaced “helplessness”)	95	• Describes a similar state as “helplessness” but one that is less extreme • The group believed this to be highly relevant to sound-based treatments that many felt can give people direct “control” over their tinnitus • One participant explained that sound-based therapy literally allowed him to “turn his tinnitus off.” • One subgroup felt that “sense of control” might cover “coping” as this was more about feelings of managing tinnitus, which also would encompass impact of activities, relationships, and social life that have been removed.	*No strong views expressed*
Quality of sleep	79	• Strong feeling that sound-based therapies (as an intervention category) are directly relevant to addressing sleep complaints associated with tinnitus. • Currently, sound therapies play a major role in improving sleep. • The group acknowledged this as one of the most reported complaints associated with tinnitus. • Argued to be highly important, given the potential to have an impact on overall well-being and given its influence on a variety of other domains.	• Some felt that “quality of sleep” was perhaps secondary to “intrusiveness” and “ability to ignore.” • The group acknowledged that sleep complaints were not relevant to all people with tinnitus, and therefore, it was questioned whether this domain should be “core.” • Some felt sleep problems were more relevant to the acute/initial phase of tinnitus and therefore maybe not appropriate for the Core Outcome Domain Set which should be relevant to both short- and long-term symptoms.
Tinnitus intrusiveness	100	• Broad coverage of tinnitus impact (e.g., can cover aspects of sleep, listening, conversation, etc.) • Captures the emotional impact of tinnitus where “tinnitus awareness” does not	One participant questioned whether “tinnitus awareness” would be more important, given that it is the “root” of tinnitus intrusiveness.

*Note*. Votes represent the % of the 19 participants who agreed that these outcome domains should be included in the Core Outcome Domain Set.

#### Psychology-based outcome domains

Twenty-four outcome domains were taken to the face-to-face meeting (Table 3). After discussion and voting, five outcomes were recommended as the Core Outcome Domain Set for early-phase clinical trials of psychology-based interventions: “acceptance of tinnitus,” “mood,” “negative thoughts/beliefs,” “sense of control,” and “tinnitus intrusiveness.” Meeting votes and reasons supporting their inclusion are described in Table 5. Supplementary File 4 gives reasons for setting aside the remaining 19 outcome domains.

**Table 5.** Meeting Votes and Comments in Favor and Against Explaining the Reasons for Recommending the Five Outcome Domains for Evaluating Psychology-Based Interventions.

Outcome domains	Vote	Comments in favor	Comments against
Acceptance of tinnitus	84	• Some stated that acceptance is an important starting point from where the person with tinnitus can start to move on. • Others felt “acceptance of tinnitus” was more important than “sense of control.”	Some felt this was a more “passive” domain that does not accurately reflect a reduction of the impact of/ distress caused by tinnitus.
Mood	100	• The group made a strong recommendation that the experience of “anxiety” and “depressive symptoms” should be added to the concept definition of “mood.”	*No strong views expressed*
Negative thoughts/ beliefs	79	*No strong views expressed*	• Some felt that this is more a process in the therapy than an outcome measure. • Some suggested this was more relevant to some psychological treatments than to others.
Sense of control	84	• The group observed that “sense of control” is particularly about feeling in control over the impact of tinnitus, perhaps as a consequence of mastering more positive coping strategies. • A construct that covers many aspects relating to tinnitus • Considered most important when symptoms are severe (e.g., sleep difficulties) • One patient felt that this is an “active” domain (unlike acceptance) that can represent a strong motivator for a patient to use a treatment.	• Some felt that applying coping techniques was more important than developing a sense of control. • The definition was considered to be too broad. • It could be encapsulated by other outcome domains. • Some participants disliked the term *control*, as this term is not well aligned with psychological treatment (i.e., tinnitus cannot be switched off).
Tinnitus intrusiveness	95	• The definition should describe in more detail in which way tinnitus can be intrusive. For this group, that meant impact on social life, impact on work, impact on relationships, impact on individual activities, difficulties getting to sleep, and quality of sleep.	*No strong views expressed*

*Note*. Votes represent the % of the 19 participants who agreed that these outcome domains should be included in the Core Outcome Domain Set.

#### Pharmacology-based outcome domains

Seventeen outcome domains were taken to the face-to-face meeting (Table 3). Only “tinnitus intrusiveness” and “tinnitus loudness” were recommended as the final set of core outcomes for early-phase drug trials. Meeting votes and reasons supporting their inclusion are described in Table 6. Supplementary File 5 gives reasons for setting aside the remaining outcome domains.

**Table 6.** Meeting Votes and Comments in Favor and Against, Explaining the Reasons for Recommending the Two Outcome Domains for Evaluating Pharmacology-Based Interventions.

Outcome domains	Vote	Comments in favor	Comments against
Tinnitus intrusiveness	100	• The group felt that “intrusiveness” captures aspects of tinnitus that are more relevant than “loudness” alone. • “Tinnitus intrusiveness” is related to “loudness” but is distinct from it. It is a target for developing a tinnitus cure based on pharmacology. • Comment indicated that this is a relatively broad construct that could be sensitive to the impact of tinnitus in a variety of areas of life (quality of life).	• A few participants believed “intrusiveness” is a subdomain of loudness (i.e., you cannot have intrusiveness without loudness). • The concept of intrusiveness may be problematic to explain consistently across different languages and cultures.
Tinnitus loudness	100	• “Tinnitus loudness” is all about the sensation of the sound. It is the direct target for drug treatments. Fix this and you fix everything else. • The group considered this to be a “semiobjective” measure and therefore reliable and critical to include alongside the more “subjective” domains. • The group felt that “loudness” needs to be measured alongside with intrusiveness as they interrelate but are separate.	Some acknowledged that a change in loudness may not always reflect a tangible benefit on the patient’s life.

*Note*. Votes represent the % of the 16 participants who agreed that these outcome domains should be included in the Core Outcome Domain Set.

### Final Voting

**Figure 3.**
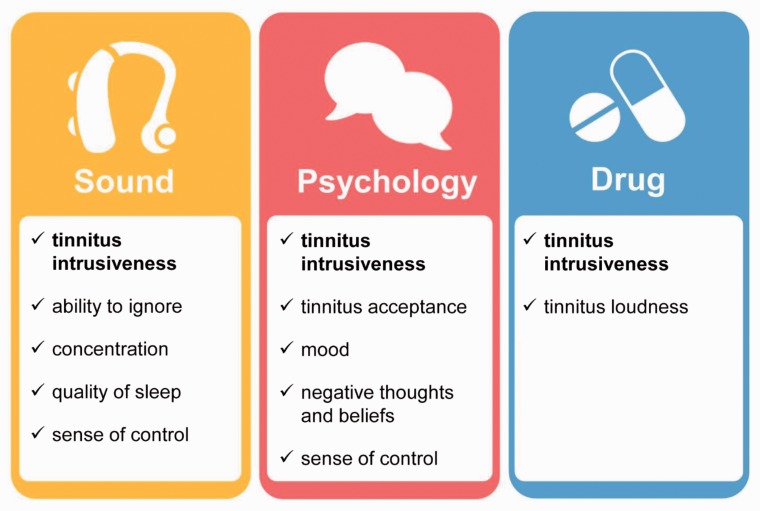
Graphic illustrating the COMiT’ID recommendations for Core Outcome Domain Sets for each family of interventions widely available for chronic subjective tinnitus in adults.

When the five outcome domains for sound-based interventions were shared with the original 338 e-Delphi participants, 144 responded and 142 voted in favor (98.6%). When the five outcome domains for psychology-based interventions were shared with the original 224 e-Delphi participants, 101 responded and 100 (99.0%) voted in favor. From the original 157 e-Delphi participants in the pharmacology-based survey, 64 out of 66 (97.0%) voted in favor of tinnitus loudness and intrusiveness. Just four participants dissented by voting “disagree,” and these were followed up in an e-mail exchange to understand their reason for voting in this way. One voter highlighted the importance of assessing and reporting adverse effects, and one considered the drug-based standard to be too small. Two dissenting voices raised concerns that differed fundamentally from the intended purpose of the project; one argued for research on the causes of tinnitus, and the other argued for a focus on “diagnostic imaging.” Figure 3 summarizes the COMiT’ID study recommendations for Core Outcome Domain Sets for chronic subjective tinnitus in adults.

## Discussion

Core outcome domain sets to be included in clinical trials of interventions for adult chronic subjective tinnitus were identified and agreed upon, for sound- (five domains), psychology- (five domains), and pharmacology-based (two domains) interventions, respectively. Participants agreed tinnitus intrusiveness was relevant and critical no matter what tinnitus intervention was being evaluated. This is an important finding because this outcome could provide a point of comparison across *any* clinical trial of a tinnitus intervention. Sense of control provides another point of comparison, at least being relevant for sound- and psychology-based approaches.

Although the three Core Outcome Domain Sets all promote intervention-related benefits, this should not downgrade the importance of assessing and reporting intervention-related harms (e.g., Ioannidis et al., 2004). In particular, discussion around the pharmacology-based standard explicitly acknowledged the importance of reported adverse events as a minimum requirement expected by the regulatory authorities.

The Core Outcome Domain Set are intended to represent a *minimum reporting standard*, chosen to be clinically meaningful and with an expectation of change as a result of intervention. Nevertheless, investigators may always be free to include additional outcomes that are important to the participant group or intervention of interest, as long as the core sets are assessed and reported as well. For example, in a trial testing cognitive behavioral therapy as an intervention for those with sleep-related difficulties, it would be justifiable to select “quality of sleep” as the primary outcome despite it not being in the core set selected for psychology-based interventions, as long as the core set were all assessed as secondary outcomes.

### Strengths and Limitations in the Development Process

The COMiT’ID study represents the first time that large numbers of the international tinnitus community have made a consensus-based decision about good practice in clinical trial design and reporting. Not only is this initiative unique in the field of tinnitus, it is exceptional in the field of *adult* hearing health care, because to our knowledge, no other Core Outcome Domain Set exists in this area. There was no compelling rationale for constraining the therapeutic benefits of the three different intervention approaches to be identical. The votes at each round, the discussion at the consensus meeting, and the final voting rounds were all conducted independently at each point in the study. A more detailed comparative analysis of the e-Delphi voting is reported in a separate article (Hall et al., 2018b). Ultimately, the minimum reporting standards chosen for each intervention approach comprised a number of unique outcome domains not selected for the other two intervention approaches. We feel that this result confirmed our decision to conduct parallel Delphi surveys, but the process might equally have identified a common set of outcome domains across all three intervention approaches. The result was not biased by the procedure. The final three sets of recommendations that emerged from the consensus decision-making process comprised outcome domains individually chosen according to those patients’ needs that are most directly addressed by each intervention approach.

Tinnitus is not necessarily unique because other health-related conditions are also treated by a range of intervention approaches. Chronic pain is one good example as it can be managed using approaches that include medications, psychology-based approaches, physical therapy, and complementary therapies. The IMMPACT group (Initiative on Methods, Measurement, and Pain Assessment in Clinical Trials) called to address this challenge by developing a core set of outcome domains that should be considered in *all* clinical trials of interventions for chronic pain, with further outcome domains to be added depending on the nature of the intervention and population to whom the intervention is targeted (Turk & Dworkin, 2004). Conceptually speaking, the COMiT’ID study is similar in its approach, but the benefit is that COMiT’ID identified from a single study what should be the common minimum standard for all trials and what should be the intervention-specific outcome domains. A number of other recent initiatives have similarly developed minimum reporting standards that are specific to a particular intervention strategy in cases where a broad range of intervention options exist. For example, the COMMENT group (Consensus group on Outcome Measures Made in pediatric Enteral Nutrition clinical Trials) separated recommendations for outcome domains relevant to clinical trials on preventing acute diarrhea from those relevant to treating the symptoms (Karas et al., 2015). Similarly, an international group interested in vaccination communication identified three different outcome domains based on whether the intended purpose of the communication was to inform or educate, to remind, or to engage the community (Kaufman et al., 2017). These examples illustrate how collective stakeholder responses prioritize different outcome domains for different types of interventions, and how minimum reporting standards need to be sensitive to those differences.

The Core Outcome Domain Set has been developed using robust methodology in accordance with recommendations from the COMET initiative (Kirkham et al., 2016). Recruitment exceeded its target, with wide representation across stakeholder groups and satisfactory retention from Round 1 to Round 3 of the e-Delphi survey. Retention rate met the 80% criterion for what is generally deemed satisfactory in the development of a Core Outcome Domain Set (Williamson et al., 2017). The purposive involvement of health-care users with tinnitus and the requirement for each selected outcome domain to have reached 70% consensus within *all* stakeholder groups avoids any risk that the outcome domain recommendations are biased in favor of clinicians and researchers. Moreover, the final voting step ensured that a wider group of international stakeholders were given the opportunity to express an opinion about the recommended common standards. In this respect, the Core Outcome Domain Set represents a major advancement on the earlier consensus statement for tinnitus intervention outcome measurement made by just 29 attendees at Tinnitus Research Initiative meeting in Regensburg (Langguth et al., 2007). We note that all of these original attendees were invited, and many did participate in the current development process. We therefore strongly believe the present recommendations are representative of the priorities and views shared by the majority of the tinnitus community.

As far as possible, it is important for Core Outcome Domain Sets to incorporate an *international* perspective (Williamson et al., 2017). Despite purposive sampling, there were still relatively low numbers of participants from Africa, Asia, and South America. There are a number of possible explanations for these limiting geographical biases. First, they reflect known biases in tinnitus clinical trial activity (Hall et al., 2016). Second, the study materials were produced in English and as a consequence would have reduced participation from countries where English is not widely spoken. With respect to the face-to-face meetings, participation from distant countries was made difficult by the choice of U.K. locations (Sheffield and London), and although European Union funding was available to support participants from across Europe, it still limited participation from elsewhere. To counteract this, the more international community of e-Delphi participants were invited to evaluate and endorse the Core Outcome Domain Set recommendations in a final vote once reports of the meetings were shared. This step achieved a lower response rate (45%) in final voting but an overwhelming support in favor (98%). Comments were actioned, where possible. For example, our recommendations highlight the importance of assessing and reporting adverse effects despite it not reaching the threshold for consensus, and we invite investigators to consider assessing other relevant domains in addition to the minimum standard.

### Implication of the Standards for Complex Interventions and Novel Interventions

With the exception of tinnitus intrusiveness, the differing composition of the three Core Outcome Domain Sets demonstrates how selection of outcome domains cannot be generalized from one family of interventions to another. For example, while “sense of control” was judged to be critical and important for evaluating sound- and psychology-based interventions, for pharmacology-based interventions, it failed even to reach the prespecified consensus criteria in the e-Delphi Round 3 voting. Faced with complex interventions (such as those combining sound and psychological components of therapy), or novel intervention strategies (such as neuromodulation), a good starting point would be to consider whether any components of the three existing standards are applicable. Certainly, “tinnitus intrusiveness” is a good candidate because it has been shown to be relevant to at least three different classes of intervention. Investigators may wish to consider additional outcome domains that are directly applicable to the intervention of interest. In such cases, there may be value in measuring outcome domains that are unrepresented in the current standard. For example, for early-phase trials evaluating neuromodulation-based interventions, it may be appropriate to measure neural activity to demonstrate intervention-related change at the neurophysiological level.

### Future Research Directions

Tinnitus can affect people in many different ways, but if the lived experience of tinnitus and the major intervention strategies are equivalent across cultures, then the Core Outcome Domain Set recommendations should be generalizable across countries, irrespective of whether citizens from that country participated in the development process. However, there is a lack of published information describing the lived experience of tinnitus in different countries because most of the published literature is limited to the United Kingdom, the United States, Germany, and Sweden (Hall et al., 2018a). Our study indicates that such cross-cultural issues warrant further investigation as a matter of priority.

The next step for the COMiT initiative is to identify “how” the outcomes in each Core Outcome Domain Set should be measured by making evidence-based decisions about which instrument best measures each outcome domain. This will involve three further pieces of work: first to define more explicitly the concepts and constructs underpinning each of the selected outcome domains, second to search for all possible available instruments, and third to identify those that have acceptable construct validity and other clinimetric properties (De Vet, Terwee, Mokkink, & Knol, 2011). When it comes to selecting measurement instruments, the logical consequence of the three different Core Outcome Domain Sets is that a single instrument will *not* meet the minimum common standard across sound-, psychology-, and pharmacology-based interventions, unless it is for tinnitus intrusiveness alone. We acknowledge that this position will challenge the generally accepted dogma that a single measurement instrument can “do the job equally well” in all tinnitus trials. Until there can be evidence-based recommendations about choice of measurement instruments, investigators would be advised in the interim to at least select instruments that purport to measure each outcome domain either as a subscale of a multidomain questionnaire instrument or a single-item numeric rating scale. Once there is an understanding about how the outcome domains should be measured, the next step is for investigators to implement these standardized sets so that there will be a common point of comparison for efficacy results across different studies evaluating the same tinnitus intervention. This will improve transparency and the ability to compare and combine future studies with greater ease. Once evidence-based recommendations are available to inform decisions about how to best measure the selected outcome domains, we aspire to conduct an observational cohort study at 7 years following publication of the recommendations. This study will evaluate uptake of the core outcomes in clinical trials, other research designs, and in systematic reviews to review the state of the field, and if necessary to understand challenges and barriers to uptake.

## Conclusions

Meta-analysis in a systematic review is possible only when outcome measures are adequately homogenous (Clarke & Williamson, 2016). It is therefore strongly advocated that all clinical trials, other research designs, and systematic reviews use these Core Outcome Domain Sets. Nevertheless, it is important to appreciate that while these minimum reporting standards should *always* be measured in *every* clinical trial (at least before and after the intervention), investigators should not necessarily feel compelled to specify them as the primary end points, and they are free to add other outcomes as they wish relevant to the specific aims and target population of their research. The recommendations are intended to provide a framework for greater compatibility across clinical trials, not to stifle individual preferences. We wish to avoid potential misunderstandings of the purpose of the Core Outcome Domain Set, which may inadvertently limit uptake and implementation.

## Supplemental Material

Supplemental Material1 - Supplemental material for The COMiT’ID Study: Developing Core Outcome Domains Sets for Clinical Trials of Sound-, Psychology-, and Pharmacology-Based Interventions for Chronic Subjective Tinnitus in Adults Click here for additional data file. Supplemental material, Supplemental Material1 for The COMiT’ID Study: Developing Core Outcome Domains Sets for Clinical Trials of Sound-, Psychology-, and Pharmacology-Based Interventions for Chronic Subjective Tinnitus in Adults by Deborah A. Hall, Harriet Smith, Alice Hibbert, Veronica Colley, Haúla F. Haider, Adele Horobin, Alain Londero, Birgit Mazurek, Brian Thacker, Kathryn Fackrell and for the Core Outcome Measures in Tinnitus (COMiT) initiative in Trends in Hearing

## Supplemental Material

Supplemental Material2 - Supplemental material for The COMiT’ID Study: Developing Core Outcome Domains Sets for Clinical Trials of Sound-, Psychology-, and Pharmacology-Based Interventions for Chronic Subjective Tinnitus in Adults Click here for additional data file. Supplemental material, Supplemental Material2 for The COMiT’ID Study: Developing Core Outcome Domains Sets for Clinical Trials of Sound-, Psychology-, and Pharmacology-Based Interventions for Chronic Subjective Tinnitus in Adults by Deborah A. Hall, Harriet Smith, Alice Hibbert, Veronica Colley, Haúla F. Haider, Adele Horobin, Alain Londero, Birgit Mazurek, Brian Thacker, Kathryn Fackrell and for the Core Outcome Measures in Tinnitus (COMiT) initiative in Trends in Hearing

## Supplemental Material

Supplemental Material3 - Supplemental material for The COMiT’ID Study: Developing Core Outcome Domains Sets for Clinical Trials of Sound-, Psychology-, and Pharmacology-Based Interventions for Chronic Subjective Tinnitus in Adults Click here for additional data file. Supplemental material, Supplemental Material3 for The COMiT’ID Study: Developing Core Outcome Domains Sets for Clinical Trials of Sound-, Psychology-, and Pharmacology-Based Interventions for Chronic Subjective Tinnitus in Adults by Deborah A. Hall, Harriet Smith, Alice Hibbert, Veronica Colley, Haúla F. Haider, Adele Horobin, Alain Londero, Birgit Mazurek, Brian Thacker, Kathryn Fackrell and for the Core Outcome Measures in Tinnitus (COMiT) initiative in Trends in Hearing

## Supplemental Material

Supplemental Material4 - Supplemental material for The COMiT’ID Study: Developing Core Outcome Domains Sets for Clinical Trials of Sound-, Psychology-, and Pharmacology-Based Interventions for Chronic Subjective Tinnitus in Adults Click here for additional data file. Supplemental material, Supplemental Material4 for The COMiT’ID Study: Developing Core Outcome Domains Sets for Clinical Trials of Sound-, Psychology-, and Pharmacology-Based Interventions for Chronic Subjective Tinnitus in Adults by Deborah A. Hall, Harriet Smith, Alice Hibbert, Veronica Colley, Haúla F. Haider, Adele Horobin, Alain Londero, Birgit Mazurek, Brian Thacker, Kathryn Fackrell and for the Core Outcome Measures in Tinnitus (COMiT) initiative in Trends in Hearing

## Supplemental Material

Supplemental Material5 - Supplemental material for The COMiT’ID Study: Developing Core Outcome Domains Sets for Clinical Trials of Sound-, Psychology-, and Pharmacology-Based Interventions for Chronic Subjective Tinnitus in Adults Click here for additional data file. Supplemental material, Supplemental Material5 for The COMiT’ID Study: Developing Core Outcome Domains Sets for Clinical Trials of Sound-, Psychology-, and Pharmacology-Based Interventions for Chronic Subjective Tinnitus in Adults by Deborah A. Hall, Harriet Smith, Alice Hibbert, Veronica Colley, Haúla F. Haider, Adele Horobin, Alain Londero, Birgit Mazurek, Brian Thacker, Kathryn Fackrell and for the Core Outcome Measures in Tinnitus (COMiT) initiative in Trends in Hearing
