# Supplementary material for: The COMiT’ID Study: Developing Core Outcome Domains Sets for Clinical Trials of Sound-, Psychology-, and Pharmacology-Based Interventions for Chronic Subjective Tinnitus in Adults
Source: Trends Hear. 2018 Nov 29;22:2331216518814384. doi: 10.1177/2331216518814384 (PMC6277759; doi:10.1177/2331216518814384)
Supplement: Supplemental Material2 - Supplemental material for The COMiT’ID Study: Developing Core Outcome Domains Sets for Clinical Trials of Sound-, Psychology-, and Pharmacology-Based Interventions for Chronic Subjective Tinnitus in Adults [file Supplemental_Material2.pdf]

Supplementary file 2. All outcome domains which failed to reach the pre-specified consensus definition based on the e-Delphi round 3 voting. *Note that participants voted to consider 'sense of control' during the face-to-face meeting of the sound-based intervention instead of 'helplessness'.

**Sound-based interventions**	**Psychology-based interventions**	**Pharmacology-based interventions**
Active myofascial trigger points	Ability to relax	Behaviour
Adverse reaction	Active myofascial trigger points	Ability to relax
Anger	Adverse reaction	Acceptance of tinnitus
Behaviour	Anger	Active myofascial trigger points
Bodily complaints	Behaviour	Anger
Brain structure	Bodily complaints	Bodily complaints
Catastrophising	Brain structure	Brain structure
Change in sense of self	Change in sense of self	Catastrophising
Confusion	Confusion	Change in sense of self
Device usage	Conversations	Conversations
Distress from bodily sensations	Device usage	Device usage
Fat metabolism	Distress from bodily sensations	Distress from bodily sensations
Fear	Fat metabolism	Fat metabolism
Feeling tired	Feeling tired	Fear
Frequency of occurrence of tinnitus episodes	Frequency of occurrence of tinnitus episodes	Feeling tired
Gene expression	Gene expression	Frequency of occurrence of tinnitus episodes
Guilt	Guilt	Gene expression
Ill health	Ill health	Guilt
Impact on relationships	Irrational beliefs	Helplessness (lack of control)
Irrational beliefs	Joyful	Ill health
Irritable	Lack of perceived support 'nobody understanding experience'	Impact on relationships
Joyful	Listening	Irrational beliefs
Lack of perceived support 'nobody understanding experience'	Loss of appetite	Irritable
Loss of appetite	Loss of peace	Joyful
Loss of peace	Monitoring	Lack of perceived support 'nobody understanding experience'
Monitoring	Neck mobility	Listening
Mood	Neck pain	Loss of appetite
Neck mobility	Need for knowledge	Loss of peace
Neck pain	Nervous	Monitoring
Need for knowledge	Neural activity	Mood
Negative thoughts/beliefs	Neuroendocrine hormones	Neck mobility
Nervous	Oxidative stress	Neck pain
Neural activity	Pain	Need for knowledge

Neuroendocrine hormones	Pharmacodynamics	Negative thoughts/beliefs
Oxidative stress	Pharmacokinetics	Nervous
Pain	Seeking support	Neural activity
Pharmacodynamics	Self-harm	Neuroendocrine hormones
Pharmacokinetics	Sexual difficulties	Oxidative stress
Seeking support	Support from family and friends	Pain
Self-harm	Teeth clenching	Pharmacodynamics
*Sense of control	Tinnitus awareness	Pharmacokinetics
Sexual difficulties	Tinnitus location	Seeking support
Suicidal thoughts	Tinnitus loudness	Self-harm
Support from family and friends	Tinnitus pitch	Sense of control
Teeth clenching	Tinnitus quality	Sexual difficulties
Tinnitus location	Tinnitus unpleasantness	Suicidal thoughts
Tinnitus loudness	Treatment satisfaction	Support from family and friends
Tinnitus pitch	Upset	Teeth clenching
Tinnitus quality	Withdrawal from treatment in the clinical trial	Tinnitus awareness
Tinnitus-related thoughts		Tinnitus location
Upset		Tinnitus pitch
Withdrawal from treatment in the clinical trial		Tinnitus quality
Worries/concerns		Tinnitus-related thoughts
		Upset
		Withdrawal from treatment in the clinical trial
		Worries/concerns
