# Supplementary material for: The COMiT’ID Study: Developing Core Outcome Domains Sets for Clinical Trials of Sound-, Psychology-, and Pharmacology-Based Interventions for Chronic Subjective Tinnitus in Adults
Source: Trends Hear. 2018 Nov 29;22:2331216518814384. doi: 10.1177/2331216518814384 (PMC6277759; doi:10.1177/2331216518814384)
Supplement: Supplemental Material3 - Supplemental material for The COMiT’ID Study: Developing Core Outcome Domains Sets for Clinical Trials of Sound-, Psychology-, and Pharmacology-Based Interventions for Chronic Subjective Tinnitus in Adults [file Supplemental_Material3.pdf]

Supplementary file 3. Comments in favour and against, explaining the reasons for setting aside those outcome domains which failed to reach the 70% consensus in the face-to-face meeting for sound-based interventions. Votes represent the % of the 19 participants who agreed that these outcome domains were not critical to be measured in every clinical trial for sound-based tinnitus interventions (threshold ≥30%). *Note that participants voted to replace 'helplessness' by the concept 'sense of control' during this face-to-face meeting.

Sound-based exclusions	Vote	Comments in favour	Comments against
Ability to relax	95	*No strong views expressed.*	Some felt this was covered by sleep outcome domains.
Acceptance of tinnitus	89	*No strong views expressed.*	• Some perceived this as a 'passive' outcome that does not reflect a reduction in the tinnitus symptoms.<br>• One participant mentioned she was never going to 'accept' her tinnitus, but that did not mean she could not cope with it.<br>• Sense of control was felt to cover elements of acceptance.
Annoyance	89	*No strong views expressed.*	• For some, 'annoyance' is associated with to 'ability to ignore' since if one can successfully ignore tinnitus, then it is not so annoying.<br>• One participant felt 'annoyance' trivialised the impact of tinnitus.
Anxiety	89	• Some felt that it is important for the COS to cover the emotional impact of tinnitus	• As with 'depressive symptoms', could be related to other domains e.g. 'coping', 'sense of control', 'treatment satisfaction' and 'intrusiveness'.<br>• Some felt this domain was too niche/ clinically focussed and therefore not relevant to all people with tinnitus.<br>• Some felt this symptom could not be changed directly through sound therapy.
Conversations	89	• Important to measure tinnitus impact on hearing ability.<br>• One subgroup wanted 'tinnitus intrusiveness' to encompass aspects of holding a conversation.<br>• One participant argued that this was a prominent issue for him.	• Not a prominent issue for all patients.<br>• Difficult to differentiate from outcomes related to hearing loss.<br>• Could be covered by intrusiveness.
Coping	47	Measuring coping could indicate the patient's progress (e.g. high levels of coping techniques required in early	• Could be covered by aspects of 'sense of control'.<br>• Confusion about how coping 'techniques' are relevant to a sound-based treatment. It was felt that the 'technique' would be the action

		stages vs. fewer coping techniques needed as the patient progresses).	of using the sound and, in this context, to measure this would not make sense.<br>• The group agreed that sound-based treatments do not always directly employ 'techniques'.<br>• Overlap with 'ability to ignore' and 'concentration'.
Depressive symptoms	89	• Some felt that it is important for the COS to cover the emotional impact of tinnitus.<br>• One participant argued that sound treatment can alleviate depressive symptoms.	• As with 'anxiety, could be covered by other domains e.g. 'coping', 'sense of control', 'treatment satisfaction' and 'intrusiveness'<br>• Some felt this domain was too niche/ clinically focussed and therefore not relevant to all.<br>• Some felt this symptom could not be changed with sound therapy.
Difficulties getting to sleep	89	The group acknowledged that this is a critical complaint, but not for all people with tinnitus.	• One subgroup felt that 'quality of sleep' was more important than 'getting to sleep' – a more holistic measure of sleep problems.<br>• One subgroup felt that difficulties was covered by quality of sleep. Argued that it is about the right amount of sleep rather than difficulties per se.<br>• Difficulties getting to sleep can also be caused by other health conditions, not related to the tinnitus.<br>• Not every person with tinnitus has difficulties getting to sleep.
Frequency of occurrence of tinnitus episodes	95	One participant argued that this is an indicator of tinnitus perception and source. Treatments may perform differently depending on this.	• Many others argued the type of tinnitus is not relevant to measuring the effect of a treatment.<br>• This domain could inform subgrouping of participants within a study but it is not seen as a meaningful outcome.
*Helplessness (lack of control)	100	*No strong views expressed.*	One subgroup placed 'helplessness' in their top 6, but there was a concern that 'helplessness' is concerned with deep suffering which is relevant only to some people with tinnitus.
Impact on individual activities	95	*No strong views expressed.*	Could be covered by 'coping' or 'tinnitus intrusiveness'.
Impact on social life	95	*No strong views expressed.*	Could be covered by 'coping' or 'tinnitus intrusiveness'.
Impact on work	95	*No strong views expressed.*	Could be covered by 'coping' or 'tinnitus intrusiveness'.

Listening	89	• Important to measure tinnitus impact on hearing ability.<br>• One subgroup wanted 'tinnitus intrusiveness' to encompass aspects of listening.	• Not a prominent issue for all people with tinnitus.<br>• Difficult to differentiate from outcomes related to hearing loss.<br>• Could be covered by 'tinnitus intrusiveness'.
Tinnitus awareness	100	• Sensitive outcome to sound-based therapy.	• Seen as the 'root domain' of tinnitus intrusiveness and other domains (e.g. a reduction in awareness would mean reduced intrusiveness) and so could be covered by 'tinnitus intrusiveness'.<br>• Does not capture the emotional impact/distress that tinnitus causes.<br>• For some, this was simply considered a 'baseline' state that does not necessarily call for treatment.<br>• 'Tinnitus intrusiveness' is potentially more sensitive to change.
Tinnitus unpleasantness	95	*No strong views expressed.*	Not as important as 'tinnitus intrusiveness'.
Treatment satisfaction	68	• This outcome was considered sufficiently broad to cover a range of therapeutic devices.<br>• It is important for people with tinnitus to be satisfied with the device they are prescribed in order for them to be motivated to use it (potentially an indicator for adherence/ compliance).	• May be coloured by factors not related to the treatment, such as a grumpy clinician etc. Moreover, 'treatment satisfaction' was felt not so suitable for early evaluation of a novel therapy (i.e. a clinical trial) because it measures satisfaction with the overall therapeutic process, not the sound device per se.<br>• Satisfaction could be achieved without effectiveness.<br>• Satisfaction is something to measure later down the line.
