# Supplementary material for: The COMiT’ID Study: Developing Core Outcome Domains Sets for Clinical Trials of Sound-, Psychology-, and Pharmacology-Based Interventions for Chronic Subjective Tinnitus in Adults
Source: Trends Hear. 2018 Nov 29;22:2331216518814384. doi: 10.1177/2331216518814384 (PMC6277759; doi:10.1177/2331216518814384)
Supplement: Supplemental Material4 - Supplemental material for The COMiT’ID Study: Developing Core Outcome Domains Sets for Clinical Trials of Sound-, Psychology-, and Pharmacology-Based Interventions for Chronic Subjective Tinnitus in Adults [file Supplemental_Material4.pdf]

Supplementary file 4. Comments in favour and against, explaining the reasons for setting aside those outcome domains which failed to reach the 70% consensus in the face-to-face meeting for psychology-based interventions. Votes represent the % of the 19 participants who agreed that these outcome domains were not critical to be measured in every clinical trial for psychology-based tinnitus interventions (threshold ≥30%).

**Psychology-based exclusions**	**Vote**	**Comments in favour**	**Comments against**
Ability to ignore	95	*No strong views expressed.*	• Some patients are able to cope with their tinnitus without trying to ignore it.<br>• Some psychological treatments specifically aim not to ignore the tinnitus.<br>• Could be covered by 'tinnitus intrusiveness'.
Annoyance	74	*No strong views expressed.*	*No strong views expressed.*
Anxiety	95	• Identified as a major focus for psychology-based treatments.<br>• When a person is anxious, it has a big impact on life.	• If 'anxiety' means a clinically meaningful anxiety disorder, then this definition applies to a very limited number of people with tinnitus.<br>• The more everyday experience of anxiety could be captured by the outcome domain 'mood'.
Catastrophising	74	*No strong views expressed.*	*No strong views expressed.*
Concentration	89	*No strong views expressed.*	• The group noted that there might be other reasons for lack of concentration other than tinnitus.<br>• One subgroup argued this might not be very sensitive to the effects of a psychological intervention.<br>• Some considered this to be similar to 'tinnitus intrusiveness'.
Coping	37	*No strong views expressed.*	Improving on a coping scale (as in using different coping strategies) does not always mean that the person is improving.
Depressive symptoms	95	When a patient is depressed, it has a big impact on life.	• If 'depressive symptoms' means a clinically meaningful depression disorder, then this definition applies to a very limited number of people with tinnitus.<br>• The more everyday experience of depression could be captured by the outcome domain 'mood'.

Difficulties getting to sleep	69	• The group identified that this is often a major focus for psychology-based intervention.<br>• One participant mentioned that sleep is fundamental to general health.	• The group noted that not everyone has ‘difficulties getting to sleep’, and so it is not a prominent issue for all people with tinnitus.<br>• Could be covered by ‘tinnitus intrusiveness’.
Fear	74	*No strong views expressed.*	*No strong views expressed.*
Helplessness (lack of control)	74	*No strong views expressed.*	*No strong views expressed.*
Impact on individual activities	95	*No strong views expressed.*	Could be covered by ‘tinnitus intrusiveness’.
Impact on relationships	74	*No strong views expressed.*	Could be covered by ‘tinnitus intrusiveness’.
Impact on social life	95	• The group acknowledged that tinnitus has a major impact on social activities (e.g. can cause isolation).<br>• One participant felt this was important because one’s social life is essential for personal well-being.	Could be covered by ‘tinnitus intrusiveness’.
Impact on work	95	*No strong views expressed.*	Could be covered by ‘tinnitus intrusiveness’.
Irritable	74	*No strong views expressed.*	*No strong views expressed.*
Quality of sleep	74	*No strong views expressed.*	The group noted that not everyone has poor ‘quality of sleep’, and so it is not a prominent issue for all people with tinnitus.
Suicidal thoughts	74	*No strong views expressed.*	*No strong views expressed.*
Tinnitus-related thoughts	74	*No strong views expressed.*	*No strong views expressed.*
Worries/concerns	74	*No strong views expressed.*	*No strong views expressed.*
