# Supplementary material for: The COMiT’ID Study: Developing Core Outcome Domains Sets for Clinical Trials of Sound-, Psychology-, and Pharmacology-Based Interventions for Chronic Subjective Tinnitus in Adults
Source: Trends Hear. 2018 Nov 29;22:2331216518814384. doi: 10.1177/2331216518814384 (PMC6277759; doi:10.1177/2331216518814384)
Supplement: Supplemental Material5 - Supplemental material for The COMiT’ID Study: Developing Core Outcome Domains Sets for Clinical Trials of Sound-, Psychology-, and Pharmacology-Based Interventions for Chronic Subjective Tinnitus in Adults [file Supplemental_Material5.pdf]

Supplementary file 5. Comments in favour and against, explaining the reasons for setting aside those outcome domains which failed to reach the 70% consensus in the face-to-face meeting for pharmacology-based interventions. Votes represent the % of the 16 participants who agreed that these outcome domains were *not* critical to be measured in every clinical trial for pharmacology-based tinnitus treatments (threshold ≥30%). *Note that participants voted to replace 'anxiety' and 'depressive symptoms' by the concept 'mood' during this face-to-face meeting.

**Pharmacology-based exclusions**	**Vote**	**Comments in favour**	**Comments against**
Ability to ignore	88	*No strong views expressed.*	• 'Ability to ignore' was considered much less ambitious than 'Intrusiveness'. 'Ability to ignore' merely suggests helping people to manage, 'intrusiveness' suggests seeking a cure.<br>• The concept could be interpreted differently by different people.
Adverse reaction	100	Agreed important to always measure for drug trials.	Adverse events are already a necessity by the regulatory authorities for any properly conducted double-blinded randomised controlled pharmacological trial, and so on that basis the group felt that it was pointless to include as a COS.
Annoyance	88	• Concept of annoyance can be easily explained and interpreted.<br>• Can have an impact on a variety of activities (e.g. social interaction)	• Some considered this too 'mild' or 'trivial'.<br>• The group felt 'annoyance' could be covered by other domains (e.g. 'intrusiveness').<br>• Some believed it to be too subjective and personality-dependent.
*Anxiety	88	People with tinnitus are often in a state of anxiety, which is separate from depressive symptoms, but may be related.	• 'Anxiety' is a secondary consequence of tinnitus.<br>• It can be treated by anxiolytics, not a direct target for tinnitus drugs.<br>• Both anxiety and depressive symptoms could be captured together as feelings related to mood. The definition of 'mood' indicates a sense of well-being rather than having a psychiatric classification.
Concentration	88	*No strong views expressed.*	• Concentration problems are not specific to tinnitus.<br>• Some felt this was covered adequately by 'tinnitus intrusiveness'.
Confusion	100	*No strong views expressed.*	Too general, may not necessarily be specific to tinnitus.
Coping	88	*No strong views expressed.*	• Coping techniques felt to be less relevant to pharmaceutical interventions.

*Depressive symptoms	88	People with tinnitus often experience depressive symptoms, which is separate from anxiety, but may be related	• 'Depressive symptoms' are a secondary consequence of tinnitus.<br>• It can be treated with antidepressants, not a direct target for tinnitus drugs.<br>• Both 'depressive symptoms' and 'anxiety' could be captured together as feelings related to mood, since they are more about having a sense of well-being than having a psychiatric classification.
Difficulties getting to sleep	100	*No strong views expressed.*	• Difficulties getting to sleep can be part of a hierarchy of sleep problems that is better captured by 'quality of sleep'. Quality of sleep is more encompassing.<br>• Not all people with tinnitus have problems with sleep and a good core outcome will be relevant to everyone.
Impact on individual activities	31	• Recognises the World Health Organisation ICF framework (i.e. activity limitations) and is an indicator of well-being.<br>• May be less influenced by personality type (introvert vs extrovert).<br>• Some felt it could be sensitive to change in the short term (i.e. during a clinical trial)	*No strong views expressed.*
Impact on social life	88	Recognises the World Health Organisation ICF framework (i.e. social participation) and is an indicator of well-being.	• Tinnitus interventions can only partly influence this outcome. It may be more to do with the number of social interactions the individual has.<br>• May be influenced by extrovert more than introvert personality type.<br>• Some doubted that people with tinnitus could show a change on this parameter over a short time period (i.e. during a clinical trial).
Impact on work	88	*No strong views expressed.*	Not applicable to those who are in education, retired, or otherwise not working
Mood (replaced 'anxiety' and 'depressive symptoms')	75	• The group noted that regulatory bodies typically seek evidence of quality of life benefit in clinical trials, so 'mood' could be an indicator of therapeutic benefit.	• The definition of 'mood' is too unspecific.<br>• The impact of 'mood' on quality of life can be captured by 'intrusiveness'.<br>• 'Mood' is a secondary consequence of tinnitus. It implies emotional issues that may not be specific to the tinnitus.

			• It can be treated with other drugs, not a direct target for tinnitus drugs.<br>• Could be more useful as a stratification tool when interpreting data from clinical trials.
Quality of sleep	62	• 'Quality of sleep' captures all different aspects of tinnitus-related sleep complaints.<br>• Disruption of sleep quality is described as a major complaint associated with tinnitus.<br>• Some suggested that poor sleep quality could directly modulate tinnitus intrusiveness and loudness so it is therefore important to understand changes in these aspects.	• Not all people with tinnitus have problems with sleep and a good core outcome will be relevant to everyone.<br>• The group felt that inclusion of 'quality of sleep' in the COS may weaken the power of the trial because it is not common to all people with tinnitus.
Tinnitus unpleasantness	100	*No strong views expressed.*	• The group had doubts that a drug therapy could change the degree of 'pleasantness' (i.e. believed unlikely to change tinnitus quality or pitch).<br>• There were mixed feelings about 'unpleasantness' since what is deemed to be unpleasant is quite individual and may be more driven by personality.<br>• Individual interpretations of the construct could make it hard to come up with a reliable measure.
Treatment satisfaction	88	*No strong views expressed.*	This is not specific to the tinnitus.
